# Clonal evolution in tyrosine kinase inhibitor-resistance: lessons from *in vitro*-models

**DOI:** 10.3389/fonc.2023.1200897

**Published:** 2023-06-13

**Authors:** Meike Kaehler, Pia Osteresch, Axel Künstner, Stella Juliane Vieth, Daniela Esser, Marius Möller, Hauke Busch, Inga Vater, Malte Spielmann, Ingolf Cascorbi, Inga Nagel

**Affiliations:** ^1^ Institute of Experimental and Clinical Pharmacology, University Hospital Schleswig-Holstein, Campus Kiel, Kiel, Germany; ^2^ Medical Systems Biology Group, University of Lübeck, Lübeck, Germany; ^3^ Institute of Cardiogenetics, University of Lübeck, Lübeck, Germany; ^4^ Institute of Clinical Chemistry, University Hospital Schleswig-Holstein, Kiel, Germany; ^5^ Institute of Human Genetics, University Hospital Schleswig-Holstein, Kiel, Germany; ^6^ Institute of Human Genetics, University Hospital Schleswig-Holstein, Lübeck, Germany

**Keywords:** chronic myeloid leukemia, drug resistance, imatinib, nilotinib, *PTPN11*, *PDGFRB*, *NRAS*, *KRAS*

## Abstract

**Introduction:**

Resistance in anti-cancer treatment is a result of clonal evolution and clonal selection. In chronic myeloid leukemia (CML), the hematopoietic neoplasm is predominantly caused by the formation of the BCR::ABL1 kinase. Evidently, treatment with tyrosine kinase inhibitors (TKIs) is tremendously successful. It has become the role model of targeted therapy. However, therapy resistance to TKIs leads to loss of molecular remission in about 25% of CML patients being partially due to BCR::ABL1 kinase mutations, while for the remaining cases, various other mechanisms are discussed.

**Methods:**

Here, we established an *in vitro*-TKI resistance model against the TKIs imatinib and nilotinib and performed exome sequencing.

**Results:**

In this model, acquired sequence variants in *NRAS*, *KRAS*, *PTPN11*, and *PDGFRB* were identified in TKI resistance. The well-known pathogenic *NRAS* p.(Gln61Lys) variant provided a strong benefit for CML cells under TKI exposure visible by increased cell number (6.2-fold, p < 0.001) and decreased apoptosis (-25%, p < 0.001), proving the functionality of our approach. The transfection of *PTPN11* p.(Tyr279Cys) led to increased cell number (1.7-fold, p = 0.03) and proliferation (2.0-fold, p < 0.001) under imatinib treatment.

**Discussion:**

Our data demonstrate that our *in vitro*-model can be used to study the effect of specific variants on TKI resistance and to identify new driver mutations and genes playing a role in TKI resistance. The established pipeline can be used to study candidates acquired in TKI-resistant patients, thereby providing new options for the development of new therapy strategies to overcome resistance.

## 1 Introduction

Despite tremendous advances within the last decades, therapy failure is still a significant burden in anti-cancer therapy. Tumor cells tend to escape chemotherapy by clonal evolution and selection of resistant subclones, resulting in a relapse in therapy. Next-generation sequencing aims to find promising candidate variants in resistant cancer cell sublines. Such approach may further be helpful for molecular tumor boards to adapt the targeted therapy regimen for each patient (1).

The myeloproliferative syndrome chronic myeloid leukemia (CML) became a role model for effective and successful targeted therapy. CML is a rare neoplasm, mainly caused by reciprocal translocation t(9;22)(q34;q11), resulting in the formation of the *BCR::ABL1* fusion gene (2). In many cases, it is successfully treated using tyrosine kinase inhibitors (TKIs), especially the 2-phenylaminopyrimidine imatinib, which binds to the BCR::ABL1 kinase and, thereby, prevents phosphorylation of downstream targets (3). Although overall 10-year survival rates are high at 83%, 20 to 25% of all patients suffer from therapy failure within five years of treatment (4, 5). Second and third-generation TKIs, namely nilotinib, dasatinib, bosutinib, and ponatinib, were developed to overcome such resistances with variable success (6, 7). TKI resistance occurs either dependent or independent from BCR::ABL1 kinase alterations. The first-mentioned is predominantly caused by mutations in *BCR::ABL1* (e.g., *ABL1* p.(Tyr253His), p.(Glu255Val) or p.(Thr315Ile)) preventing binding of the TKIs to the kinase domain or by *BCR::ABL1* gene amplification and overexpression (8). For BCR::ABL1-independent resistance, several mechanisms are discussed, e.g., overexpression of drug efflux transporters, especially the ATP binding cassette (ABC) transporter family members p-glycoprotein (P-gp, ABCB1) or breast cancer resistance protein (BCRP, ABCG2), the adaption of signaling pathways or deregulation of gene expression (9, 10). In addition, genetic aberrations, e.g., trisomy 8 or mutations affecting runt-related transcription factor 1 (*RUNX1*), were shown to contribute to the progression into blast crisis or selection of TKI-resistant clones in patients (11, 12).

Besides clinical studies, *in vitro*-models can be applied to study mechanisms of drug resistance in detail. Such models are pivotal tools as findings derived from these models were successfully translated into the clinic, e.g., to predict drug efficacy and improve treatment protocols (13). Drug resistance of a tumor cell line can be acquired *in vitro* by exposure to slowly increasing anticancer drug concentrations or by pulse treatment.

Here, we used exome sequencing to study genetic variants in a TKI resistance CML *in vitro*-model. For this purpose, we established biological replicates of imatinib and nilotinib resistance. We report on sequence variants evolving in imatinib and nilotinib resistance development. Further, we investigate the influence of the candidate variants *PTPN11* p.(Tyr279Cys), *PDGFRB* p.(Glu578Gln), and *NRAS* p.(Gln61Lys) on the response to TKI treatment.

## 2 Materials and methods

### 2.1 Reagents, cell lines, and generation of resistant cells

If not indicated otherwise, chemicals and reagents were obtained from Sigma-Aldrich (Munich, Germany) or Carl Roth (Karlsruhe, Germany).

K-562 cells (RRID: CVCL_0004), established from the pleural effusion of a 53-year-old woman (14), were obtained from the German Collection of Microorganisms and Cell Cultures (DSMZ, Braunschweig, Germany). Cell maintenance, generation of biological replicates of TKI-resistant sublines, and analyses of cell line authenticity were described elsewhere (15, 16). Cells were resistant against lowIM (0.5 µM imatinib), highIM (2 µM imatinib), lowN (0.05 µM nilotinib) and highN (0.1 µM nilotinib). The concentrations were chosen to reflect the clinically typical range of estimated imatinib plasma concentration, as well as the 20-fold higher potency of nilotinib.

### 2.2 RNA and DNA extraction

Total RNA was isolated using E.Z.N.A Total RNA kit 1 (Omega bio-tek, Norcross, GA, USA). Cell line DNA was purified using Gentra Puregene Kit (Qiagen, Hilden, Germany).

### 2.3 Exome sequencing

Exome sequencing was performed using Illumina InView Human Exome Advance sequencing technology, a random-primed cDNA library, 60x coverage, and 2 x 150 bp read length at Eurofins Genomics (Ebersberg, Germany). Raw data was mapped against GRCh38. Exome data was processed similarly to Künstner et al. (17). For the detailed bioinformatic analysis, see **supplement**.

### 2.4 MiSeq

Exome sequencing data was validated using Next Generation Sequencing (NGS) SBS technology with Illumina MiSeq after PCR amplicon preparation with the Nextera XT Sequencing Kit (Illumina, San Diego USA). For this purpose, amplicons of the respective genes were generated using gene-specific primers, primer-specific annealing temperatures and MyTaq DNA Polymerase (Meridian Bioscience, Memphis, TN, USA). (**Supplementary Table 1**). Genomic DNA from 2 µM imatinib resistant K-562 cells replicate 2, and 0.1 µM nilotinib resistant K-562 cells replicate 2 served as templates. PCR products were extracted using GeneJet Gel Extraction Kit (Thermo Fisher Scientific, Darmstadt, Germany) according to the manufacturer’s recommendations. MiSeq was performed according to the manufacturer’s protocol, as already described (18).

### 2.5 Genome-wide expression analyses

Microarrays were performed using Clariom S Arrays (Affymetrix; Thermo Fisher Scientific) as previously described (16). Briefly, RNA was isolated using miRVANA microRNA isolation kit (Thermo Fisher), and 100 ng were hybridized onto the arrays according to the manufacturer’s protocol. Further details about data processing and analysis are given in the supplement.

### 2.6 Whole-cell lysates and immunoblotting

Whole-cell lysates and immunoblotting were performed as described elsewhere (19, 20). Blots were probed with the following antibodies obtained from Santa Cruz or CST (Danvers, MA, USA): phospho-ERK: Cat# sc-7383, RRID AB_627545, 1:1000; ERK: Cat# sc-514302, RRID : AB_2571739, 1:1000; SHP2: Cat# 3397, RRID: AB_2174959, 1:1000; PDGFRβ: Cat# sc-374573, RRID: AB_10990921, 1:100; pan-RAS: Cat# sc-166691, RRID: AB_2154229, 1: 200; GAPDH: Cat# sc-47724, RRID: AB_627678, 1:2000; anti-rabbit: Cat# 926-32211, RRID: AB_621843; Cat# 926-926-68071, RRID: AB_10956166; anti-mouse: Cat# 926-32210, RRID: AB_621842, Cat# 926-680707, RRID: AB_10956588; all 1:10,000, LiCOR (Bad Homburg, Germany). Primary antibodies were diluted in Intercept/TBS blocking solution (LiCOR) supplemented with 0.2% Tween-20, secondary antibodies were diluted in TBS supplemented with 0.1% Tween-20. Total protein staining was performed using Revert 700 Total Protein Stain Solution according to the manufacturer’s protocol (LiCOR). Densitometry was performed using Empiria Studio 1.2 (LiCOR).

### 2.7 Inhibition assay

PTPN11 phosphatase activity was blocked using the allosteric inhibitor RMC-4550 (ProbeChem, Shanghai, China). For this purpose, 1 x 10^6^ cells per sample were seeded onto 12 well plates and incubated with 1.5 µM RMC-4450 for 3 h in a cell culture incubator. Subsequently, cells were collected, and immunoblotting was performed as described above.

### 2.8 Cloning

The coding regions of *PTPN11* (NM_002834.5) and *NRAS* (NM_002524.5) were amplified using cDNA from highIM-R2 and highN-R2 cells. *PDGFRB* coding plasmid was obtained from Sino Biological (NM_002609.3, HG10514-G, Eschborn, Germany). The amplicons were cloned into the pSelect-puromycin-mcs vector (Sigma-Aldrich) using the CloneAmp HiFi PCR premix (Takara) with gene-specific primers and primer-specific annealing temperatures (**Supplementary Table S1**) including the restriction enzymes BamHI and NcoI/NheI (NEB), cloning enhancer and the In-Fusion HD Kit (Takara). *PDGFRB* p.(Glu578Gln) was inserted using Q5 site-directed mutagenesis kit (NEB) using the primers *PDGFRB*_Glu578Gln_F and *PDGFRB*_Glu578Gln_R at 60°C annealing temperature and 3 min elongation time according to the manufacturer’s protocol (**Supplementary Table S1**). Sequence identity was confirmed using Sanger sequencing.

### 2.9 Transient and stable transfection

Transient transfection was performed using nucleofection and the nucleofector 2 b device (Lonza, Cologne, Germany). 2 x 10^6^ cells were transfected with 5 or 10 µg of the respective plasmid or empty vector control for plasmid transfection or 100 nM Ambion Silencer Select s11524 or negative control #1 for siRNA-mediated knockdown of *PTPN11*. 24 h after transfection, cells were seeded onto respective cell culture plates to analyze cellular fitness followed by 24-48 h exposure to 2 µM imatinib or 100 nM nilotinib or used for expression analyses as described elsewhere. After incubation time, cells were subducted for subsequent cellular fitness assays as described below. Stably transfected cells were generated by selecting puromycin-resistant cells after 4 weeks of exposure to 1 µg/ml puromycin (Invivogen, Toulouse, France).

### 2.10 Cellular fitness assays

Cellular fitness was analyzed as previously described (16, 18, 19). Briefly, cell numbers were obtained by trypan blue staining, WST-1 (Sigma-Aldrich), Caspase Glo 9 Assay (Promega), Bromodeoxyuridine proliferation assay (Merck, Darmstadt, Germany), and MKI ELISA Kit (MyBioSource, San Diego, CA, USA) according to the manufacturers’ recommendations. Data was analyzed by normalizing TKI-treated to non-treated samples, followed by statistical analyses as described below. For analyses of total cell number, proliferation, and apoptosis during the development of imatinib resistance, 0.5 x 10^6^ cells/ml were seeded into cell culture flasks and exposed to 0.1 µM imatinib for 21 days. Cells were counted and cultivated dependent on the cell density. After 21 days, Ki-67 expression and caspase 9 activity were measured as described above. The analyses of 0.2 and 0.3 µM imatinib were performed accordingly.

### 2.11 Statistical analysis

Unless not stated otherwise, statistical analysis was performed using one-way ANOVA, Dunnett’s test and/or student’s t-test and the GraphPad prism software (Version 8.0 for Windows, San Diego California, USA).

## 3 Results

### 3.1 Genetic analyses reveal large differences between biological replicates of imatinib and nilotinib resistance

**Figure 1:**
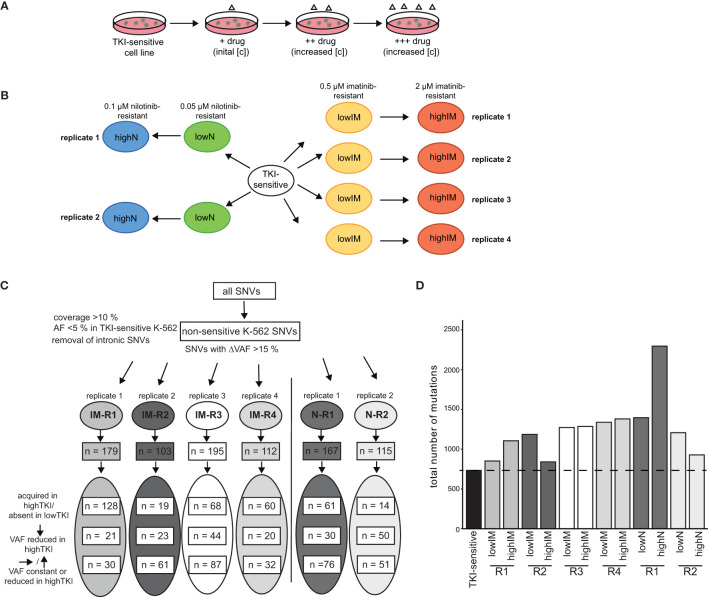
Analyses of genetic aberrations in TKI-resistant sublines. **(A)** Schematic representation of the generation of TKI-resistant cell lines *in vitro*. TKI-sensitive cells were exposed to an initial drug concentration (IM: 0,1 µM; N: 0.01 µM). When the cellular proliferation rate was restored, the drug concentration was stepwise increased (IM: 0.3, 0.5, 0.7. 1.0, 1.5 and 2 µM; N: 0.02, 0.05, 0.07 and 0.1 µM). **(B)** Overview of the TKI-resistant sublines used for the present study: Four imatinib-resistant sublines, resistant against low (0.5) and high (2 µM) imatinib, and two nilotinib-resistant sublines, resistant against low (0.05) and high (0.1 µM) nilotinib, were analyzed and compared to TKI-sensitive K-562 cells. **(C)** Analysis pipeline for the TKI-resistant cell lines. Using a coverage of >10%, the removal of SNVs already present in TKI-sensitive cells (VAF < 0.05) and removal of deep intronic SNVs, SNVs with a difference in the variant allele frequency (VAF) >15% between TKI-sensitive and resistant cell lines were obtained. The numbers indicate the SNVs clustered into variants acquired in highTKI/absent in lowTKI, variants with reduced VAF in highTKI and variants with constant or reduced VAF the high TKI-resistant cell lines compared to low TKI-resistant cells. **(D)** Total number of mutations in the TKI-resistant sublines. IM, imatinib; N, nilotinib; TKI, tyrosine kinase inhibitor; R, replicate.

To analyze clonal evolution in TKI resistance, imatinib and nilotinib-resistant sublines derived from TKI-sensitive K-562 cells were established by step-wise exposure to increasing TKI concentrations (**Figure 1A**). Cell lines developing resistance against 0.5 µM imatinib (lowIM) or 2 µM imatinib (highIM), as well as 0.05 µM nilotinib (lowN) or 0.1 µM nilotinib (highN) were obtained generating four biological replicate cell lines of imatinib and two of nilotinib resistance (**Figure 1B**). Subsequently, genetic variants in these twelve TKI-resistant sublines were analyzed by exome sequencing and compared to TKI-sensitive K-562 cells.

First, non-intronic single nucleotide variants (SNVs) exclusively present in TKI-resistant cells were identified by excluding SNVs present in TKI-sensitive K-562 (VAF < 0.05) and applying a ΔVAF > 15% in the TKI-resistant sublines compared to TKI-sensitive cells. The number of variants differed between 103 and 195 in the TKI-resistant sublines (**Figure 1C**, **Supplementary Table S2**). For IM-R1 and IM-R4, the majority of SNVs, 128 and 60, respectively, were newly acquired in highIM, whereas for IM-R2 and IM-R3, as well as in N-R1 and N-R2, the majority of SNVs were already present in the respective lowIM or lowN sublines (IM-R2: 61, IM-R3: 87, N-R1: 76, N-R2: 51, **Figure 1C**). The total number of SNVs differed between the biological replicates of TKI resistance but increased compared to TKI-sensitive cells in all TKI-resistant cell lines (**Figure 1D**). However, for N-R1, a strong increase in the total SNV number was detected in highN compared to lowN, while in highIM-R2, as well as in highN-R2, the total number of SNVs was lower compared to lowIM-R2 or lowN-R2 cells, respectively (**Figure 1D**). To generate insight into the mutational processes, we determined the mutational signatures (COSMIC, https://doi.org/10.11093/nar/gky1015) of the variants that were acquired in the TKI-resistant sublines (VAF < 5% in TKI-sensitive K-562, △VAF > 15% between TKI-sensitive and -resistant K-562 cells). In all sublines, the signatures of unknown etiology, SBS40, showed the strongest signal (**Supplementary Figure S1**).

As proteins interact in protein-protein-interaction (PPI) networks, this can be analyzed using network-based approaches, such as network propagation. Following this idea, mutations in single genes (protein) can be viewed as ‘heat sources’ in a PPI network. This heat can diffuse through the rest of the network using an iterative process until a steady state is reached. Proteins close to the mutated protein get higher propagation scores than distant proteins following the biological assumption that proteins underlying similar phenotypes tend to interact with one another (21, 22). Accordingly, the protein-protein interaction network of acquired variants was determined

**Figure 2:**
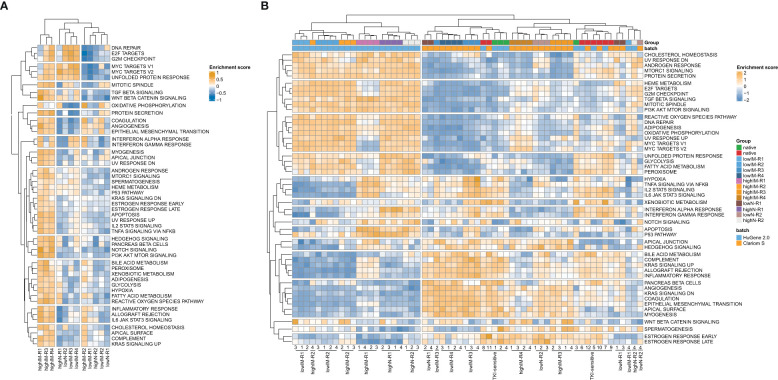
Pathway networks in TKI resistance. **(A)** Network propagation of SNVs with ΔVAF>0.15 in the TKI-resistant sublines compared to TKI-sensitive cells. **(B)** Scaled enrichment scores of the gene set variation analysis (GSVA) in TKI resistance. Genome-wide gene expression data was obtained from Clariom S arrays, as well as the dataset GSE203442. IM, imatinib; N, nilotinib; R, replicate.

Three clusters were revealed for the resistant cell lines and 14 clusters for gene sets with highIM-R1, -R3 and -R4 being a distinct cluster separate from the other tested resistant sublines (**Figure 2A**). To compare the network propagation with gene expression data, genome-wide expression analyses of the TKI-resistant cell lines and gene set variation analyses were performed [(16), **Figure 2B**]. The resulting pattern of enriched pathways was highly similar to one of the protein-protein interaction network derived from the mutational pattern (**Figure 2A**].

### 3.2 *In vitro*-TKI resistance is associated with pathogenic variants in well-known oncogenes

**Figure 3:**
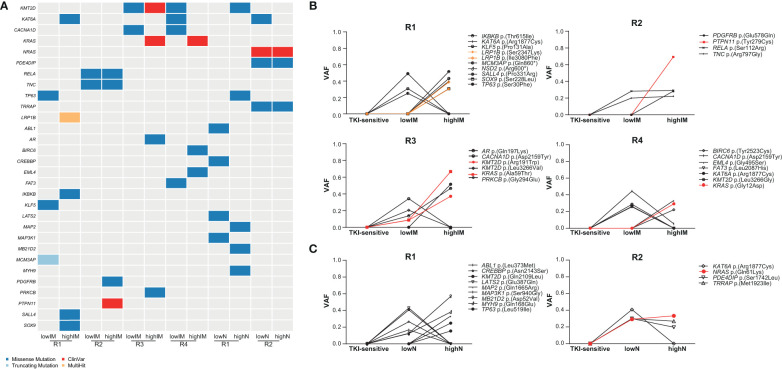
Identification of putative candidate driver variants in TKI resistance. **(A)** Oncoplot showing potential candidate driver mutations in the TKI-resistant cell lines obtained from association with a list of mutational oncogenes from Martínez-Jiménez et al. (23). Dark blue: missense mutations, light blue: truncating mutations, Red: ClinVar mutation, orange: multihit variants. **(B, C)** Proportion of SNVs in the TKI-resistant replicate cell lines shown as variant allele frequencies (VAFs). Red indicates ClinVar, orange multihit variants. IM, imatinib; N, nilotinib; R, replicate.

To identify potential driver mutations in the TKI-resistant sublines, acquired SNVs (with the respective AF ≤ 5% in sensitive K-562 cells) were compared to a list of 568 mutational cancer driver genes previously published by Martínez-Jiménez et al. (23) (**Figure 3A**, **Table 1**). Between two and five of the detected mutations in each TKI-resistant cell line were mapped to genes from the mutational cancer driver gene list. Among the acquired variants were the well-known pathogenic *RAS*-family mutation *KRAS* (KRAS proto-oncogene, GTPase) p.(Ala59Thr) (ClinVar ID: 12581; lowIM-R3: 8.7%, highIM-R3: 66.6%) in IM-R3, *KRAS* p.(Gly12Asp) in IM-R4 (ClinVar ID: 12582; lowIM-R4: absent; highIM-R4: 29%, **Figures 3A, B**), as well as *NRAS* (*NRAS* proto-oncogene, GTPase) p.(Gln61Lys) (ClinVar ID: 73058; lowN: 29.2%, highN: 33.3%, **Figures 3A, C**) in N-R2. Further, two pathogenic *KMT2D* (lysine methyltransferase 2D) variants p.(Leu3266Val) and p.(Arg191Trp) (ClinVar ID: 449928) were acquired in IM-R3 (lowIM-R3: 9%, highIM-R3: 37%, **Figures 3A, B**). Moreover, *PTPN11* (protein tyrosine phosphatase non-receptor 11) p.(Tyr279Cys) was detected in IM-R2 (ClinVar ID: 13328; lowIM-R2: absent; highIM-R2: 69%, **Figures 3A, B**). In this cell line, the previously unknown *PDGFRB* (platelet-derived growth factor receptor beta) variant p.(Glu578Gln) was also detected (lowIM-R2: absent, highIM-R2: 28%, **Figures 3A, B**, **Table 1**). The gain of these SNVs likely explains the development of TKI resistance in the respective cell lines.

**Table 1:** Variants in tumor driver genes acquired in TKI resistance.

Gene symbol	Classification	Sample	ClinVar ID, rs-number, COSMIC ID	CADD score	VAF
***ABL1***	chr9: 130873012C>A NM_007313: c.1117C>A p.(Leu373Met)	lowN-R1		25.2	0.2648
***AR***	chrX: 67545735C>A NM_000044: c.589C>A p.(Gln197Lys)	highIM-R3		22.2	0.4655
***BIRC6***	chr2: 32482454A>G NM_016252: c.7568A>G p.(Tyr2523Cys)	highIM-R4		25.5	0.2222
***CACNA1D***	chr3: 53811368G>T NM_000720: c.6508G>T p.(Asp2170Tyr)	lowIM-R3 lowIM-R4		29	0.3429 0.4444
***CREBBP***	chr16: 3728619A>G NM_004380: c.6428A>G p.(Asn2143Ser)	lowN-R1	COSV52114804	10.68	0.4066
***EML4***	chr2: 42303119G>A NM_019063: c.1690G>A p.(Gly564Ser)	highIM-R4		32	0.3333
***FAT3***	chr11: 92799273T>A NM_001367949: c.6260T>A p.(Leu2087His)	lowIM-R4		26.2	0.2526
***IKBKB***	chr8: 42322352C>T NM_001556: c.1844C>T p.(Thr615Ile)	highIM-R1		21.1	0.5152
***KAT6A***	chr8: 41932591G>A NM_006766: c.5635G>A p.(Arg1879Cys)	highIM-R1 lowIM-R4 lowN-R2	rs772414652, COSV55902233	31	0.3967 0.2611 0.4081
***KLF5***	chr13: 73062263C>G NM_001730: c.664C>G p.(Pro222Ala)	lowIM-R1	COSV100890535	18.09	0.3077
***KMT2D***	chr12: 49041444T>A NM_003482: c.6326T>A p.(Gln2109Leu)	highN-R1		23.6	0.2481
***KMT2D***	chr12: 49037560G>C NM_003482: c.9796G>C p.(Leu3266Val)	lowIM-R3 lowIM-R4		23.2	0.2043 0.2875
***KMT2D***	chr12: 49054080G>A NM_003482: c.571G>A p.(Arg191Trp)	highIM-R3	449928, rs1555198522, COSV56467834	29.6	0.371
***KRAS***	chr12: 25227349C>T NM_033360: c.175C>T p.(Ala59Thr)	highIM-R3	12581, rs121913528, COSV55499283, COSV55796966	24.2	0.6667
***KRAS***	chr12: 25245350C>T NM_033360: c.35C>T p.(Gly12Asp)	highIM-R4	12582 rs121913529, COSV55497369 COSV55497419, COSV55497479	23.7	0.2927
***LATS2***	chr13: 20988621C>G NM_014572: c.1159C>G p.(Glu387Gln)	lowN-R1		17.95	0.4286
***LRP1B***	chr2: 140487651A>T NM_018557: c.9209A>T p.(Ile3070Lys)	highIM-R1		26.7	0.3881
***LRP1B***	chr2: 140598785G>A NM_018557: c.7040G>A p.(Ser2347Phe)	highIM-R1		26.6	0.304
***MAP2***	chr2: 209710175A>G NM_001375505: c.5483A>G p.(Glu1828Arg)	highN-R1	rs1184836325	26.1	0.3333
***MAP3K1***	chr5: 56882018A>G NM_005921: c.2818A>G p.(Ser940Gly)	lowN-R1		13.86	0.1667
***MB21D2***	chr3: 192917686T>A NM_178496: c.155T>A p.(Asp52Val)	highN-R1		28.7	0.5682
***MCM3AP***	chr21: 46270451G>A NM_003906: c.2578G>A p.(Gln860*)	lowIM-R1		41	0.25
***MYH9***	chr22: 36327477G>C NM_002473: c.502G>C p.(Gln168Glu)	highN-R1		23.9	0.381
***NRAS***	chr1: 114713909C>A NM_002524: c.181C>A p.(Gln61Lys)	lowN-R2 highN-R2	73058, rs121913254, COSV54736310, COSV54743343, COSV54752117	26.4	0.2917 0.3333
***PDE4DIP***	chr1: 149009789C>T NM_001350521: c.5333C>T p.(Ser1778Leu)	lowN-R2 highN-R2		24.1	0.3 0.1961
***PDGFRB***	chr5: 150125520G>C NM_002609: c.1732G>C p.(Glu578Gln)	highIM-R2		26.3	0.283
***PRKCB***	chr16: 24113032G>A NM_002738: c.881G>A p.(Gly294Glu)	highIM-R3	rs199901715	22	0.5152
***PTPN11***	chr12: 112473023A>G NM_01330437: c.836A>G p.(Tyr279Cys)	highIM-R2	13328, rs121918456, CM021133, CM041069, COSV61009292	29.9	0.6929
***RELA***	chr11: 65660125T>A NM_021975: c.426T>A p.(Gln142His)	lowIM-R2 highIM-R2		22.4	0.2828 0.2923
***SALL4***	chr20: 51791491G>C NM_020436: c.992G>C p.(Pro331Arg)	highIM-R1	COSV53854623	26.1	0.4322
***SOX9***	chr17: 72122970C>T NM_000346: c.683C>T p.(Ser228Leu)	highIM-R1	COSV55423902, COSV55424856	30	0.3066
***TNC***	chr9: 115081787T>C NM_002160: c.2389T>C p.(Arg797Gly)	lowIM-R2 highIM-R2		25.5	0.2 0.2222
***TP63***	chr3: 189737766C>T ENST00000264731: c.89C>T p.(Ser30Phe)	lowIM-R1		26.1	0.4909
***TP63***	chr3: 189889387C>A NM_003722: c.1555C>A p.(Leu519Ile)	highN-R1	COSV53199362, COSV99289295	21	0.1538
***TRRAP***	chr7: 98955211G>A NM_001375524: c.5844G>A p.(Met1948Ile)	lowN-R2 highN-R2		24.6	0.2993 0.2685

Variants in tumor driver genes according to Martinez-Jiménez et al. (22) (VAF in TKI-sensitive K-562: < 5%, △VAF between TKI-sensitive and -resistant K-562: >15%) including the classification, sample in which the variant was acquired, CADD score v1.6 and variant allele frequency (VAF) in the TKI-resistant sublines according to GRCh38/hg38. It should be noted that the K-562 cell line is triploid. lowIM: 0.5 µM imatinib-resistant, highIM: 2 µM imatinib-resistant, lowN: 0.05 µM nilotinib-resistant, highN: 0.1 µM nilotinib-resistant K-562 cells, R1: replicate 1, R2: replicate 2, R3: replicate 3, R4: replicate 4.

As *ABL* mutations are frequently the reason for TKI failure, mutations in this gene were also taken into focus showing two variants of unknown significance p.(Leu373Met) in lowN-R1 and p.(Glu208Asp) (VAF: 7%) in highIM-R4, as well as the known pathogenic kinase-domain mutation p.(Glu274Lys), with the latter likely associated with the TKI resistance (VAF: 10%, **Figure 3A**).

### 3.3 *NRAS* p.(Gln61Lys) impairs the response to TKI treatment

**Figure 4:**
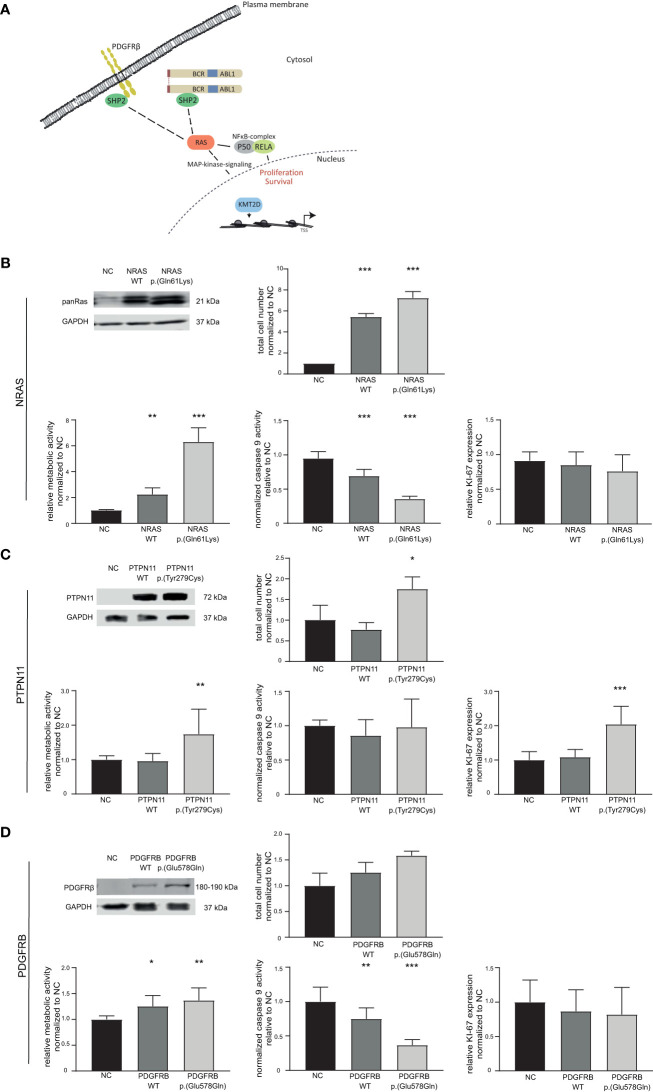
Effect of candidate variants *NRAS* p.(Gln61Lys), *PTPN11* p.(Tyr279Cys) and *PDGFRB* p.(Glu578Gln) on the response to TKI treatment. **(A)** Graphical representation of the pathways affected by variants in the candidate genes *NRAS/KRAS*, *PTPN11* (encoding SHP2), *PDGFRB* and *KMT2D*. **(B–D)** Top left: Western Blot of successful transfection of wild-type (WT) and variant into TKI-sensitive K-562 cells compared to GAPDH. Cellular fitness after WT and variant transfection and 48 h nilotinib exposure (0.1 µM) for **(B)** NRAS WT and p.(Gln61Lys), as well as imatinib exposure (2 µM) for **(C)** PTPN11 WT and p.(Tyr279Cys) and **(D)** PDGFRB WT and p.(Glu578Gln). Top right: Total cell number analyzed using trypan blue staining. Bottom left: Metabolic activity measured by WST assay. Bottom middle: Caspase 9 activity analyzed by caspase 9-Glo assay. Bottom right: Ki-67 expression to investigate cellular proliferation. Data was normalized to respective negative control (NC) and analyzed using Two-way ANOVA followed by Dunnett’s test. N = 3. Error bars indicate standard deviation. *p < 0.05, **p < 0.01, ***p < 0.001.

Presence of variants in *NRAS*, *KRAS* as well as *PTPN11*, *PDGFRB*, *RELA*, and *KMT2D* in the TKI-resistant sublines pointed to recurrent pathway changes, especially in Ras-MAP-kinase signaling (**Figure 2**; **Figure 4A**). As *NRAS* p.(Gln61Lys) is a well-known driver mutation, described in various cancer types and associated with malignancy and tumor progression, the effect of this mutation in our *in vitro*-model was analyzed to investigate whether it is solely sufficient for the development of TKI resistance and if this effect is detectable with our *in vitro*-model (**Supplementary Table S3**). To address this, TKI sensitive K-562 cells were transfected with either *NRAS* wild-type or the p.(Gln61Lys) variant. The response to nilotinib was analyzed measuring cell number, metabolic rate activity, apoptosis, and proliferation rates. Successful transfection of K-562 cells led to a 4.4-fold increase in cell number after *NRAS* WT (p < 0.001) and 6.2-fold after p.(Gln61Lys) transfection compared to the negative control (p < 0.001, **Figure 4B**). In addition, metabolic activity was increased in *NRAS* WT (1.2-fold, p = 0.002) and p.(Gln61Lys)-transfected cells (5.2-fold, p < 0.001; **Figure 4B**), while apoptosis, visible on the level of caspase 9 activation, was decreased (WT: 25%; p < 0.001; p.(Gln61Lys): 59%, p< 0.001; **Figure 4A**). However, proliferation measured by Ki-67 expression was not significantly altered between the cell lines (**Figure 4B**). A similar effect was also observed under imatinib exposure, as cell number (62%, p = 0.008) and metabolic activity (3.7-fold, p < 0.001) were increased and apoptosis was reduced after *NRAS* p.(Gln61Lys) transfection (-45%, p = 0.003), while proliferation did not significantly change (**Supplementary Figure S2**). Overall, our data demonstrate that the presence of *NRAS* p.(Gln61Lys) is solely sufficient to promote TKI resistance.

### 3.4 *PTPN11* p.(Tyr279Cys), but not *PDGFRB* p.(Glu578Gln) promote the development of imatinib resistance

Using our established *in vitro*-analysis pipeline, we focused on the *PTPN11* p.(Tyr279Cys) variant (IM-R2: VAF: 48%, **Supplementary Table S3**). Successful transfection of *PTPN11* p.(Tyr279Cys) into sensitive K-562 cells led to an increase in cell number (1.7-fold, p = 0.03), accompanied by an increase in metabolic activity (1.7-fold, p = 0.005) and proliferation (2.0-fold, p < 0.001). Nevertheless, a change in apoptosis was not observed (**Figure 4C**).

*PTPN11* p.(Tyr279Cys) is a well-known pathogenic germline variant associated with Leopard- and Noonan-syndrome (24, 25). However, this particular variant’s role in cancer and CML is widely unknown. To investigate the effect of *PTPN11* p.(Tyr279Cys) in imatinib resistance and on the context-dependent protein function, first, *PTPN11* expression was analyzed in the imatinib-resistant sublines showing no expression differences compared to TKI-sensitive K-562 cells (**Supplementary Figure S3**). As several studies indicated a loss of catalytic function for *PTPN11* p.(Tyr279Cys) (26), we hypothesized that the observed effect could be due to altered phosphatase activity. To address this, TKI-sensitive K-562 cells were exposed to the PTPN11 inhibitor RMC-4450, and the effects on Ras-MAP-kinase signaling were analyzed on the level of ERK activation. As expected, PTPN11 inhibition reduced the phosphorylation of ERK (**Supplementary Figure S4A**). To investigate if PTPN11 blockade alters the response to imatinib, an siRNA-mediated knockdown of *PTPN11* was performed to mimic reduced protein levels (**Supplementary Figure S4B**). The knockdown cells were subsequently exposed to imatinib resulting in decreased metabolic activity (-32%, p < 0.001) and BrdU incorporation (-33%, p = 0.02), while apoptosis was not altered (**Supplementary Figure S4C**). Moreover, PTPN11 inhibition was performed in the imatinib-resistant cell lines to investigate the extent of pathway addiction in these cells. Interestingly, PTPN11 inhibition only resulted in reduced ERK-phosphorylation in highIM-R2 (-1.3-fold, p = 0.04), but not in the other resistant sublines (**Supplementary Figure S4D**). The siRNA-mediated knockdown of *PTPN11* in this cell line showed a slight decrease in imatinib susceptibility, as BrdU incorporation was 1.3-fold increased (p = 0.02), while metabolic activity and apoptosis were not altered (**Supplementary Figures S4E, F**).

As a further candidate variant, we analyzed *PDGFRB* p.(Glu578Gln), as PDGFRB is a well-known target of imatinib (IM-R2: VAF: 34%, **Supplementary Table S3**). Although overexpression of *PDGFRB* p.(Glu578Gln) did not lead to a significant increase in cell number, metabolic activity was increased (WT: 1.3-fold, p = 0.01; p.(Glu578Gln): 1.4-fold, p = 0.002) and caspase 9 activity reduced after WT and p.(Glu578Gln) transfection (WT: 25%, p = 0.007; p.(Glu578Gln): 74%, p < 0.001). Analyses of proliferation measured by Ki-67 expression did not reveal significant differences (**Figure 4D**).

**Figure 5:**
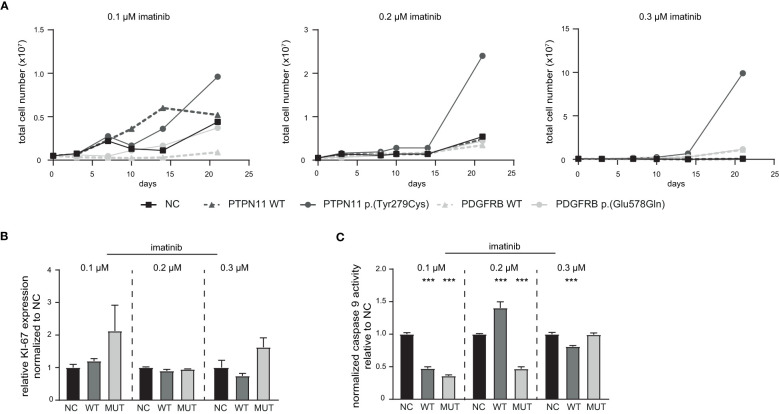
Influence of *PTPN11* p.(Tyr279Cys) and *PDGFRB* p.(Glu578Gln) on the development of imatinib resistance. Stably transfected cells expressing either *PTPN11* wild-type (WT), p.(Tyr279Cys), or *PDGFRB* WT or p.(Glu578Gln) were exposed to increasing concentrations of imatinib. **(A)** Cells were cultivated with the respective imatinib concentration and the total cell number was analyzed using trypan blue staining for 0.1, 0.2, and 0.3 µM imatinib within 21 days. Black: Negative control (NC); dark grey: PTPN11; light grey: PDGFRB; solid line: mutation; dashed line: WT. **(B)** Ki-67 expression to analyze proliferation and **(C)** Caspase 9 activity of *PTPN11* WT and p.(Tyr279Cys) transfected cells after 21 days of treatment with the respective imatinib concentration measured by caspase 9-Glo assay. Data were normalized to NC. Statistical analysis was performed using two-way ANOVA followed by Dunnett’s test. N = 3. Error bars indicate standard deviation. ***p < 0.001.

Next, stably transfected cell lines expressing either *PTPN11* WT or p.(Tyr279Cys), as well as *PDGFRB* WT or p.(Glu578Gln) were generated. These cell lines were exposed to low dose imatinib (0.1 to 0.3 µM) and the total cell number was analyzed during the development of imatinib resistance in a time-frame of 21 days (**Figure 5**). An increase in the cell number of *PTPN11* p.(Tyr279Cys)-expressing cells were detected in all tested imatinib concentrations, while the other cell lines showed no differences compared to the negative control-transfected cells (**Figure 5A**). As *PTPN11* p.(Tyr279Cys) seemed to promote an advantage for the cells during the development of imatinib resistance, proliferation and apoptosis in these cell lines after two weeks of exposure to the respective imatinib concentration was analyzed. Compared to WT and negative control-transfected cells, no significant increase in the proliferation of p.(Y279)-transfected cells was detected (**Figure 5B**). An imatinib dose-dependent effect was observed in WT-expressing cells on apoptosis (0.1 µM: -53%, p < 0.001; 0.2 µM: +40%, p < 0.001; 0.3 µM: -19%, p < 0.001), while cells harboring p.(Tyr279Cys) showed reduced apoptosis in 0.1 µM (74%, p < 0.001) and 0.2 µM imatinib-resistant cells (53%, p < 0.001), but not in resistance to 0.3 µM imatinib (**Figure 5C**).

## 4 Discussion

Tumor cells undergoing anti-cancer therapy underlie clonal evolution and selection, a major obstacle to successful treatment. In chronic myeloid leukemia, 20-25% of patients suffer from therapy failure within 5 years of TKI treatment (5). In half of the patients, mutations in the BCR::ABL1 kinase are detected, resulting in loss of TKI binding and, subsequently, resistance (4). For the other half of the patients, the resistance mechanisms are unknown. To generate insights into mechanisms of TKI resistance, an *in vitro*-TKI resistance model derived from TKI-sensitive K-562 CML cells during long-term drug exposure was established. For this purpose, biological replicates of imatinib and nilotinib resistance were generated obtaining sublines resistant against 0.5 and 2 µM imatinib, and 0.05 and 0.1 µM nilotinib, respectively. In CML patients, the imatinib plasma levels varied between 0.34 and 3.4 µM (27, 28). Thus, the imatinib concentrations of 0.5 and 2 µM were used to reflect the fluctuations. As nilotinib is known to have a 20-fold higher potency than imatinib (6), cells resistant to a maximum of 0.1 µM were used for the present study.

Large differences between the sublines were observed analyzing the mutational landscape of the TKI-resistant cell lines. The two cell lines harboring variants in the oncogene *KRAS* (highIM-R3, highIM-R4), showed a high similarity in the protein-protein interaction network compared to TKI-sensitive K-562 cells. These are likely to be addressed by the overall gained variants and are also visible in the gene expression profiles. Although the number of variants gradually increased during the development of imatinib resistance in both cell lines, in IM-R3 *KRAS* p.(Ala59Thr) accompanied by *KMT2D* p.(Arg191Trp) are likely to be crucial for resistance. In IM-R4, the combination of the well-known *KRAS* p.(Gly12Asp) mutation and a low-frequency mutation in *ABL* p.(Glu274Lys) are possibly central for resistance. Consistent with these results, *RAS* mutations are known to be tumor driver variants in myeloid neoplasia and have also been identified in CML patients with therapy failure (29, 30). However, with an incidence of only 5%, mutations in RAS seem to be a rare event in chronic myeloid leukemia (29, 31). In general, the *KRAS*-mutated cells showed a similar network propagation cluster compared to TKI-sensitive cells pointing to restored Ras-MAP-kinase signaling being the underlying resistance mechanisms.

In N-R2, the *NRAS* p.(Gln61Lys) mutation apparently occurs early during the development of nilotinib resistance. This well-known mutation occurs in 13-25% malignant melanoma and 1-6% colorectal cancer patients, but also other tumors harbor this mutation, e.g., neuroblastoma, non-small cell lung cancer or leukemia (32). However, the pattern of *NRAS* mutations in leukemia varies widely from solid tumors with a predominance for the observed p.(Gln61) (38%) and p.(Gly12) (36%) missense mutations (29, 33). The p.(Gln61Lys) mutation leads to constitutive NRAS activation. It is the reason for therapy failure, as observed, e.g., for epidermal growth factor receptor (EGFR) inhibition, which makes alternative therapies necessary (34). We found that expression of *NRAS* p.(Gln61Lys) promotes survival of CML cells under TKI exposure by reduction of apoptosis and increased cell viability, showing that this mutation is likely the main cause of resistance of our nilotinib-resistant subline N-R2. As *NRAS* p.(Gln61Lys) is already present in lowN and provides a strong benefit for the clones harboring this mutation, this indicates clonal selection and might also be a reason for the quite low overall number of mutations in this resistant subline compared to the other cell lines. Overall, these findings show that our *in vitro*-CML drug resistance model is suitable for detecting genetic aberrations promoting TKI resistance, among them pathogenic variants that have already been detected in other tumor entities. Therefore, we used the established analysis pipeline to investigate further candidate mutations.

Similar to N-R2, the overall number of acquired variants was quite low for IM-R2. Although the *PTPN11* p.(Tyr279Cys) and *PDGFRB* p.(Glu578Gln) variants were acquired in the late development of TKI resistance in highIM-R2, the allele frequencies were high in this cell line. This indicates a benefit for the clones harboring these variants. PTPN11/SHP2 is a non-receptor phosphatase involved in fine-tuning of cell signaling by binding to its adaptor proteins Grb2 and Gab1 and is considered a positive regulator of RAS signaling (35). *PTPN11* germline variants are present in 50% of patients suffering from Noonan-syndrome, an autosomal dominant disorder associated with heart failure and facial dysmorphia (25), or Leopard syndrome, a genetic disease mainly leading to heart and skin anomalies (24). In about 85% of patients, missense variants in *PTPN11* are observed, including the variant p.(Tyr279Cys), as detected in the present study. Further, somatic *PTPN11* variants occur in 34% of juvenile myelomonocytic leukemia and were also detected in other myelodysplastic syndromes, yet to a smaller extent (36). The main observed variants in *PTPN11* affect residues in the N-terminal Src homology (N-SH2) or the protein tyrosine phosphatase (PTP) domain interacting surface and likely result in a gain of function. This led to the description of PTPN11 as an atypical phosphatase with oncogenic properties, which makes PTPN11 a suitable target for cancer therapy (36, 37). It was shown that inhibition of PTPN11 might be an effective strategy to overcome NRAS-dependent resistance in neuroblastoma or KIT-induced myeloproliferative diseases (38, 39).

In the present study, a decreased imatinib response in the presence of *PTPN11* p.(Tyr279Cys) was observed with increased proliferation rates. In CML, *PTPN11* variant (NM_002834.5:c.1529A>T) p.(Gln510Leu), which is also a known variant in Noonan-syndrome (RCV001261023.1), was detected in a patient suffering from blast crisis after 10 years of TKI treatment (40). Further, it was demonstrated that PTPN11 is necessary for BCR::ABL1-induced hematologic neoplasms, as its deletion compromised induction of CML in mice (41). In a study in K-562 cells, it was shown that PTPN11 phosphorylation is induced during imatinib exposure as well as resistance and PTPN11 inhibition is able to restore TKI response (42). However, for p.(Tyr279Cys) in Leopard syndrome, a loss of catalytic activity was demonstrated (26, 43). It is likely that the observed variant in *PTPN11* results in an alteration of the phosphatase activity, which contributes to the development of TKI resistance. Nevertheless, further studies are necessary to investigate the effect of *PTPN11* p.(Tyr279Cys) in more detail. The relevance of *PTPN11* in CML is also stressed by findings on CD34+ CML stem cells harboring the pathogenic *PTPN11* p.(Gly60Val) resulting in sustained Ras-MAP-signaling pathway activation. Interestingly, the resistance in these cells could be overcome by synergistic usage of TKIs and integrated stress response inhibitors that prevent the cellular response to the external and internal stress stimuli, such as imatinib (44). This could be a very promising approach in TKI-resistant CML patients.

We detected the *PDGFRB* p.(Glu578Gln) variant in the same imatinib-resistant subline. This tyrosine kinase is a known target of imatinib and is implicated in multiple diseases, e.g., dermatofibrosarcoma protuberans or myofibromatosis (45). Several variants, but also genetic rearrangements, in *PDGFRB* were shown to be associated with TKI resistance (46). The observed p.(Glu578Gln) variant is located in the juxtamembrane portion of the protein and has not been described yet. Interestingly, it was shown that PTPN11 can suppress transformation induced by PDGFRB, suggesting a strong link between these two proteins (47). Therefore, it can be hypothesized that the two variants in *PDGFRB* and *PTPN11* can circumvent imatinib-induced BCR::ABL1 inhibition and contribute to the manifestation of TKI resistance. Thus, monitoring of variants in these genes should be performed in TKI-relapsed CML patients.

For IM-R1 and N-R1, no clear candidate driver variants were observed. Moreover, these cells clustered apart from the other cell lines in the network propagation. Potentially, the clones have a diverse mechanism of resistance which is reflected by a diverse pattern of variants. As previously mentioned, CML resistance occurs either due to BCR::ABL1-dependent or -independent mechanisms. It is widely known that especially mutations in *BCR::ABL1* affect TKI-response and lead to relapse due to the uprising of mutated clones (48). Beyond *BCR::ABL1*, variants in the epigenetic modulator DNA (cytosine-5)-methyltransferase 3A (*DNMT3A*), the polycomb group protein additional sex comb-like 1 (*ASXL1*), runt-related transcription factor 1 (*RUNX1*) and Tet methylcytosine dioxygenase 2 (*TET2*) were shown to be associated with therapy failure indicating defective epigenetic DNA regulation in TKI-resistant CML as already described for other myeloproliferative syndromes (12, 49–51). In the present study, we did not detect variants in these genes but identified several putative candidate genes that likely contribute to TKI resistance. With *NRAS*, *KRAS*, *PTPN11*, and *KMT2D*, we detected variants in genes that were shown to be mutated in low frequencies in blast crisis, but to a lower extent also in chronic phase CML (52). These findings suggest an NGS-based screening of TKI-resistant patients without BCR::ABL1 mutations to identify potential variants responsible for therapy failure. Either detected variants or the altered downstream pathways could be future therapeutic options to be targeted by synergistic approaches to overcome TKI resistance.

In conclusion, the TKI-resistant sublines newly acquired candidate driver mutations, especially the well-known *NRAS* p.(Gln61Lys), *KRAS* p.(Ala59Thr) and p.(Gly12Asp), but also *PTPN11* p.(Tyr279Cys) affected the same signaling pathway. The gain of these variants likely explains the main mechanism resistance in the respective cell lines. It shows that such models are potentially useful to get insight into mechanisms of drug resistance and to find novel tumor driver genes or novel driver mutations. This knowledge can be used to better interpret TKI resistance in patients and, vice versa, our *in vitro*-model can be used to analyze and assess mutations observed in resistant patients. This strategy can open new options for the development of new therapy strategies.

## Data availability statement

The datasets presented in this study can be found in online repositories. The whole exome sequencing data of TKI-resistant cell lines have been submitted to the European Nucleotide Archive (ENA) and is publicly available under accession number PRJEB60565. Genome-wide expression datasets are available in the GEO repository GSE227347.

## Author contributions

MK and IN conceptualized the study and designed the research. PO and SV performed the experiments. AK, DE, MM, IV, MK, and IN analyzed the data. MK and IN interpreted the data. HB, MS and IC provided methodology. MK and IN wrote the original draft. All authors read and approved the final version of the manuscript.
