# Supplementary material for: Clonal evolution in tyrosine kinase inhibitor-resistance: lessons from in vitro-models
Source: Front Oncol. 2023 Jun 13;13:1200897. doi: 10.3389/fonc.2023.1200897 (PMC10294234; doi:10.3389/fonc.2023.1200897)
Supplement: Supplementary file 1 [file DataSheet_1.pdf]

Supplementary table S1.

Overview of PCR primers and conditions for MiSeq, cloning and mutagenesis.

**Primer name**	**Genomic region (hg38)**	**Genomic orientation**	**Primer sequence 5'→3'**	**Annealing temperature (°C)**	**PCR amplicon (bp)**
**Mi-Seq**					
PTPN11_F	Chr.12: 112472962 - 112472981	**+**	TGCAAACTTCTCTACAGCCG	60	318
PTPN11_R	Chr.12: 112473280-112473261	**-**	AACCAACCACAGACAAAGCC		
PDGFRβ_F	Chr.5: 150125399-150125418	+	CCAACTTGAGTCCCCACACT	60	344
PDGFRβ_R	Chr.5: 150125743 - 150125724	-	CGAAGACCTTGGCTCAGTTG		
NRAS_F	Chr.1: 114713725 - 114713745	**+**	TGTGGTAACCTCATTTCCCCA	53	300
NRAS_R	Chr.1: 114714025-114714020	**-**	ATTGAACTTCCCTCCCTCCC		
**Cloning**					
PTPN11_pSELECT_F	Chr.12: 112419112-112419130	+	GCGTGTCGACGGATCATGACATCGCGGAGATGG	61	1812
PTPN11_pSELECT_R	Chr.12: 112486633-112486608	-	TGTCTGGCCAGCTAGTCATCTGAAACTTTTCTGCTGTTGC		
PTPN11_Y279C_seq_R	Chr.12: 112477941 - 112477922	-	CATTCACCGTGTTTTGCAGG		
PTPN11_Exon_5-6-7_seq_F	Chr.12: 112454591 - 112454613	+	GAACGGTTTGATTCTTTGACAGA		

PDGFRB_ pSELECT_F	Chr.5: 150137047-150137029	-	GCGTGTCGACGGATCATGCGGCTTCCGGGTGCG	61	3322
PDGFRB_ pSELECT_R	Chr.5: 150115763-150115787	+	TGTCTGGCCAGCTAGCTACAGGAAGCTATCCTCTGCTTCC		
PDGFRB_ Exon4_S	Chr.5: 150134950-150134931	-	AGATCACCATTCCATGCCGA		
PDGFRB_ Exon6_S	Chr.5: 150133611-150133592	-	AAAGGCCATCAACATCACCG		
PDGFRB_ Exon10_S	Chr.5: 150129864-150129844	-	GAGGAGCAGGAGTTTGAGGT		
PDGFRB_ Exon14_S	Chr.5: 150124341-150124322	-	GAAGCAAGCCCTTATGTCGG		
PDGFRB_ Exon17_S	Chr.5: 150121288-150121569	-	CGAGTCTCCAGTGCTAAGCT		
PDGFRB_ Exon21_S	Chr.5: 150118773-150118754	-	AGACTGTTGGGCGAAGGTTA		
PDGFRB_ E578Q_F	Chr.5: 150125530-150125509	-	TGACGGCCATCAGTACATCTAC	60	3322
PDGFRB_ E578Q_R	Chr.5: 150125530-150125548-	+	GAGCTCACAGACTCAATC		
NRAS_ pSELECT_F	Chr.1: 114704469 -114704496	-	GCGTGTCGACGGATCATGACTGAGTACAAACTGGTGGTCC	62	570
NRAS_ pSELECT_R	Chr.1: 114716743-114716771	+	GCCAGCTAGCCCATGTTACATCACCACACATGGCAATCCC		
pSELECT_F			CTCTCCACGCTTTGCCTGA		
pSELECT_R			TCACTGCATTCTAGTTGTGGT		

Supplementary Table S3.

Allele frequencies of candidate variants derived in TKI-resistant sublines measured by exome or MiSeq sequencing.

Gene ID	Variant	Genomic localization (hg38)	TKI-resistant cell line	Exome sequencing		Mi-seq	
				**Coverage**	**h_n**	**Coverage**	**h_n**
PTPN11	NM_01330437: c.836A>G p.(Tyr279Cys)	Chr.12:112473023	highIM-R2	124	69 %	894	48 %
PDGFRB	NM_002609: c.1732G>C p.(Glu578Gln)	Chr.5:150125520	highIM-R2	95	31 %	2331	34 %
NRAS	NM_002524: c.181C>A p.(Gln61Lys)	Chr.1:114713909	highN-R2	82	33 %	34680	37 %

Supplementary Figure S1. Somatic signatures of TKI resistant sublines. Mutational signatures of the SNVs acquired in the TKI resistant replicate cell lines (VAF <5 % in TKI-sensitive K-562, △VAF >15 % between TKI-sensitive and -resistant K-562 cells) according to COSMIC (https://doi.org/10.11093/nar/gky1015). IM: imatinib, N: nilotinib, R: replicate.

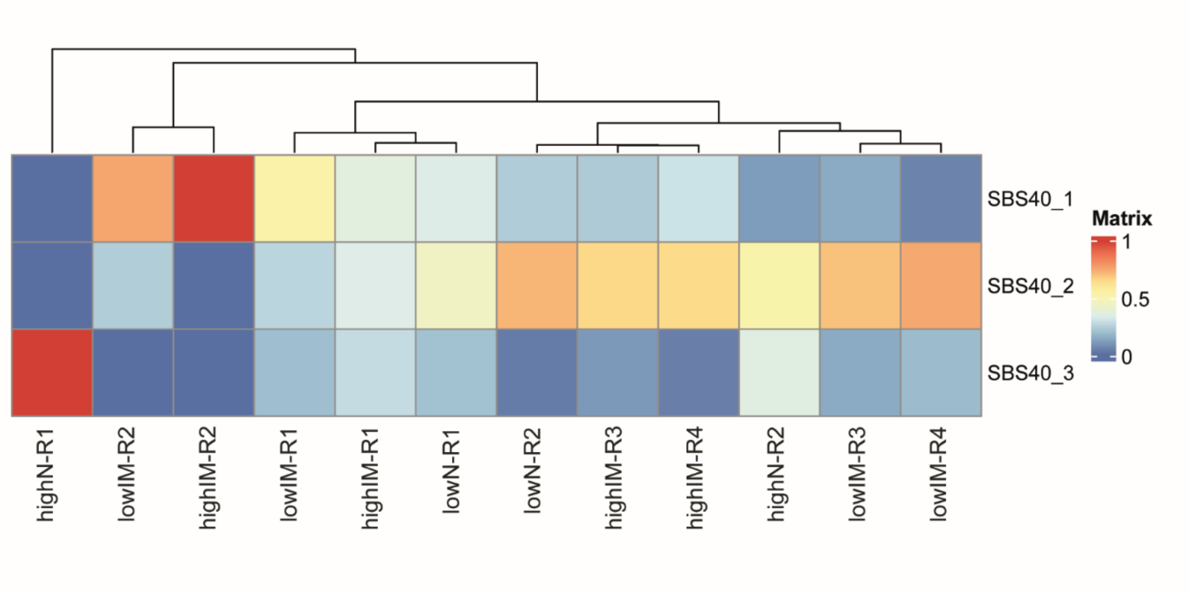

Supplementary Figure S2. NRAS p.(Gln61Lys) impairs the response to imatinib. Cellular fitness after transfection of NRAS WT and p.(Gln61Lys) followed by 48 h nilotinib exposure (0.1 µM). (**A**) Total cell number analyzed using trypan blue staining, (**B**) metabolic activity, (**C**) caspase 9 activity and (**D**) Ki-67 expression. Data were normalized to respective negative control (NC) and analyzed using Two-way ANOVA followed by Dunnett's test. N = 3. Error bars indicate standard deviation. **: $p < 0.01$, ***: $p < 0.001$.

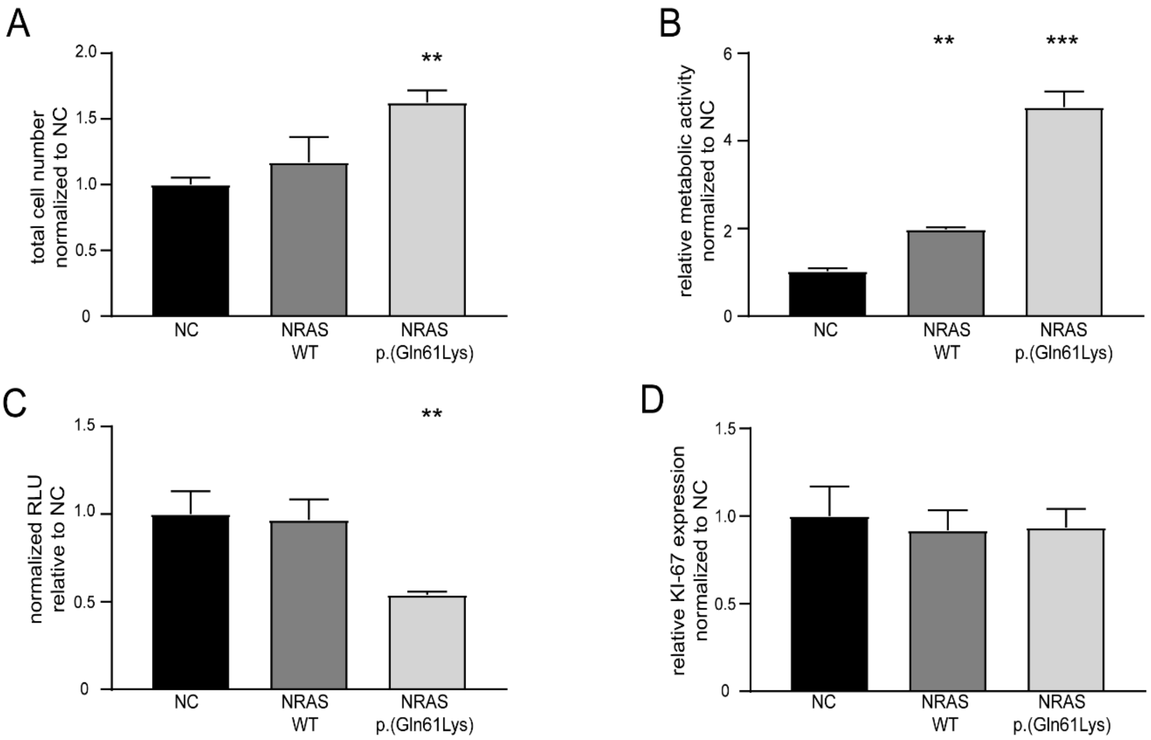

Supplementary Figure S3. Expression of PTPN11 in imatinib resistance. PTPN11 protein level (**A**) of TKI-sensitive K-562 compared to highIM sublines compared to total protein. (**B**) Densitometry of PTPN11 protein level in highIM sublines normalized to TKI-sensitive K-562 and total protein. N = 3. R1: replicate 1; R2: replicate 2; R3: replicate 3; R4: replicate 4.

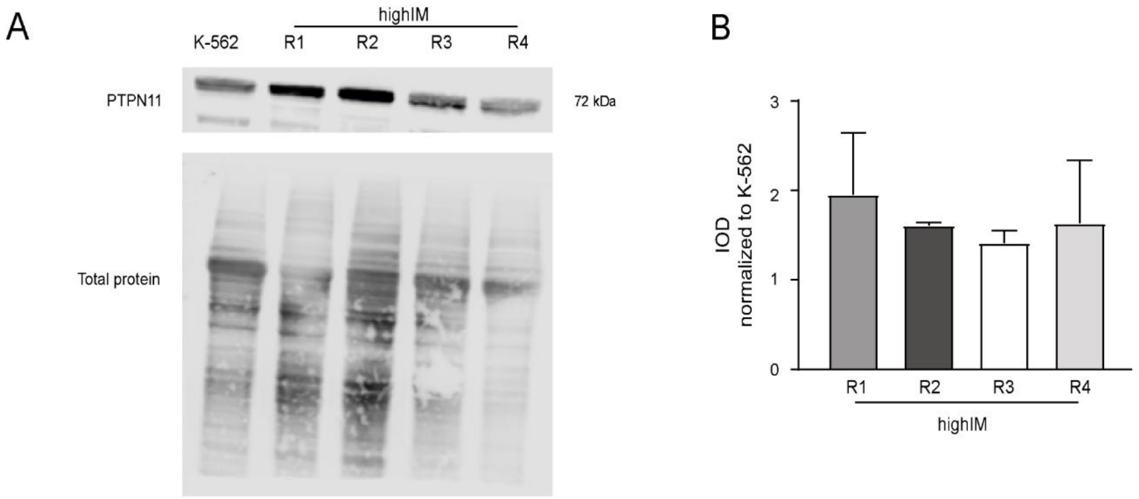

Supplementary Figure S4. Role of PTPN11 under imatinib exposure and resistance. (**A**) Phosphorylation of ERK after inhibition of PTPN11 in native K-562 cells using RMC-4550 compared to DMSO. (**B**) SiRNA-mediated knockdown of PTPN11 expression in native K-562 compared to GAPDH. (**C**) Cellular fitness of *PTPN11*-knockdown cells under exposure to 2 µM imatinib compared to negative control transfection (NC) analyzed on the level of (left) metabolic activity, (middle) caspase 9 activity and (right) BrdU incorporation. Data normalized to NC. (**D**) Phosphorylation of ERK after PTPN11 inhibition using RMC-4550 in 2 µM imatinib resistant sublines R1 to R4 including densitometry normalized to DMSO-treated cells. (**E**) Western blot of siRNA-mediated PTPN11 knockdown in highIM-R2 compared to GAPDH. (**F**) Cellular fitness of PTPN11 knockdown in highIM-R2 cells measured by (left) metabolic activity), (middle) caspase 9 activity and (right) BrdU incorporation compared to NC. Error bars indicate standard deviation. Statistical analysis was performed using two-way ANOVA followed by Dunnett's test or student's t-test. N = 3. *: $p < 0.05$; ***: $p < 0.001$. RLU: relative luminescence units.

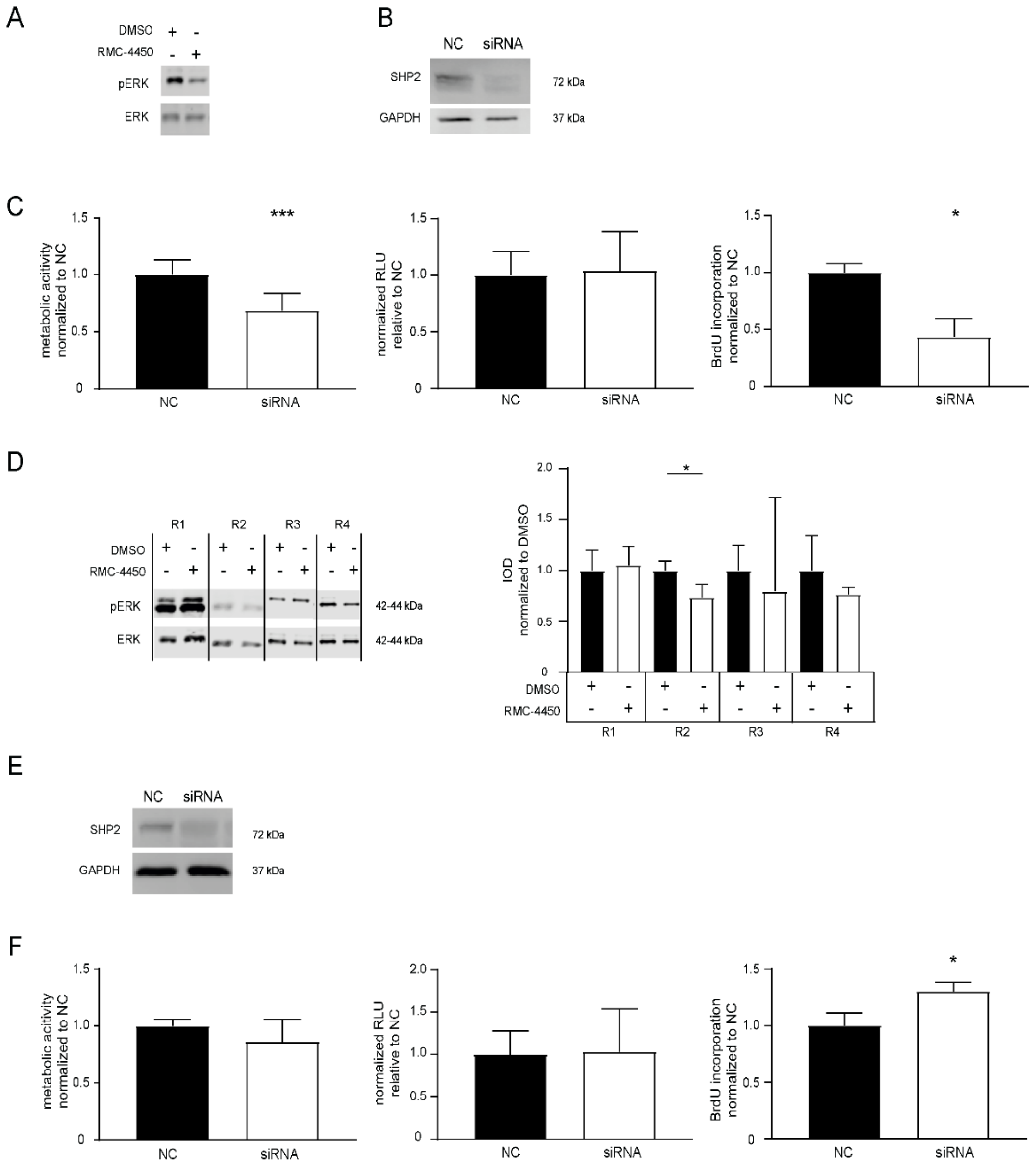
A
DMSO + -
RMC-4450 - +
pERK
ERK
B
NC siRNA
SHP2
72 kDa
GAPDH
37 kDa
C
metabolic acitivity normalized to NC
***
NC
siRNA
normalized RLU relative to NC
BrdU incorporation normalized to NC
*
D
R1 R2 R3 R4
DMSO
RMC-4450
pERK
ERK
42-44 kDa
IOD normalized to DMSO
*
R1 R2 R3 R4
E
NC siRNA
SHP2
72 kDa
GAPDH
37 kDa
F
metabolic acitivity normalized to NC
normalized RLU relative to NC
BrdU incorporation normalized to NC
*
NC
siRNA

Supplementary Methods

Exome data processing and analysis

Raw sequencing reads (*fastq* format) were trimmed (adaptor sequences and low-quality bases/reads were removed) applying fastp (v0.23.2; minimum length, 50 bp; maximum unqualified bases, 30%; trim tail set to 1)(Chen et al., 2018). Trimmed reads were mapped to GRCh38 using bwa mem2 (v2.2.1)(Vasimuddin et al., 2019) and mappings were converted into *BAM* format using Picard Tools (v2.26.10, Institute B. Picard Toolkit. GitHub Repository; 2019). Next, mate-pair information was fixed, PCR duplicates were removed, and base quality recalibration was performed applying Picard Tools, GATK (v4.2.4.1)(McKenna et al., 2010) and dbSNP v138 (Sherry et al., 2001). Single nucleotide variants (SNVs) and short insertions and deletions (indels) were identified following GATKs best practices for tumor-only somatic mutation calling. Briefly, Mutect2 (GATK)(Cibulskis et al., 2013) was applied to all *BAM* files with gnomAD variants as germline resource and the b38 exome panel from the 1000 genome project as a panel of normal, capturing the expected repertoire of germline variants to be expected in a Central European study population. Variants were annotated using Variant Effect Predictor (VEP v103, GRCh38; adding CADD v1.6, dbNSFP v4.1a, and gnomAD r3.0 as additional annotations (McLaren et al., 2016;Rentzsch et al., 2019)) and annotations were converted into MAF format using vcf2maf (v1.6.21) (doi:10.5281/zenodo.593251); coverage was extracted directly from the vcf INFO field. The top 20 frequently mutated genes (FLAGS, (Shyr et al., 2014)) and variants occurring in the TKI-sensitive K-562 cells with an allele frequency above 5% were removed from further analysis and the remaining somatic variants were filtered as follows: minimum coverage of 40, minimum alternative allele coverage of 5, minimum variant allele frequency of 15%, CADD score > 10, and only variants with a

frequency < 0.1% in 1000 genomes, gnomAD, or ExAC were considered for subsequent downstream analysis.

The effect of strong deleterious mutations (CADD1.6 phred score > 20, vaf > 0.15, TKI-sensitive K-562 variants removed) was assessed per sample using a network propagation approach with a regularized Laplacian kernel based on STRINGdb v11 protein-protein interaction network as implemented in the diffuStats R package (v1.16.0) (Picart-Armada et al., 2018;Szklarczyk et al., 2019).Mutated genes were set to 1, whereas non-mutated genes were set to 0 to model the behavior of strongly deleterious mutations (“heat source”). Network propagation was performed using a parametric method with statistical normalization (*z*-scores). Afterwards, gene set variation analysis was performed on diffusion scores with a Gaussian kernel for continuous values against HALLMARK gene set collection (minimum/maximum size of the resulting gene sets set to 10/500).

Microarray data processing and analysis

Microarray data (CEL files) was imported to R (v4.2.0) and preprocessed using robust multichip average (rma) to perform background correction and quantile normalization (oligo package v1.60.0). For the gene set variation analysis, additionally, the dataset GSE203442 (GEO database, https://www.ncbi.nlm.nih.gov/) was included in the analyses. Both data sets were combined and batch correction was performed applying an empirical Bayes framework (*ComBat* function as provided by sva v3.44.0). Next, gene set variation analysis was performed using the GSVA R package (v1.44.5, (Hanzelmann et al., 2013)) with a Gaussian kernel for continuous values against HALLMARK gene sets (R package msigdf v7.4 with minimum/maximum size of the resulting gene sets set to 10/500; MSigDB; Molecular Signatures Database (MSigDB) in a data frame; https://github.com/ToledoEM/msigdf; (Liberzon et al., 2015)).

Data handling and statistics

Exome and microarray data were imported in R and the following libraries were used for data handling: maftools (v2.12.0), tidyverse (v1.3.2), and data.table (v1.14.6). Heatmaps were generated using ComplexHeatmaps (v2.12.1). Optimal number of clusters was determined using the NbClust package (v3.0.1, Euclidean distance and kmeans clustering).

References

Chen, S., Zhou, Y., Chen, Y., and Gu, J. (2018). fastp: an ultra-fast all-in-one FASTQ preprocessor. *Bioinformatics* 34**,** i884-i890.

Cibulskis, K., Lawrence, M.S., Carter, S.L., Sivachenko, A., Jaffe, D., Sougnez, C., Gabriel, S., Meyerson, M., Lander, E.S., and Getz, G. (2013). Sensitive detection of somatic point mutations in impure and heterogeneous cancer samples. *Nat Biotechnol* 31**,** 213-219.

Hanzelmann, S., Castelo, R., and Guinney, J. (2013). GSVA: gene set variation analysis for microarray and RNA-seq data. *BMC Bioinformatics* 14**,** 7.

Liberzon, A., Birger, C., Thorvaldsdottir, H., Ghandi, M., Mesirov, J.P., and Tamayo, P. (2015). The Molecular Signatures Database (MSigDB) hallmark gene set collection. *Cell Syst* 1**,** 417-425.

Mckenna, A., Hanna, M., Banks, E., Sivachenko, A., Cibulskis, K., Kernytsky, A., Garimella, K., Altshuler, D., Gabriel, S., Daly, M., and Depristo, M.A. (2010). The Genome Analysis Toolkit: a MapReduce framework for analyzing next-generation DNA sequencing data. *Genome Res* 20**,** 1297-1303.

Mclaren, W., Gil, L., Hunt, S.E., Riat, H.S., Ritchie, G.R., Thormann, A., Flicek, P., and Cunningham, F. (2016). The Ensembl Variant Effect Predictor. *Genome Biol* 17**,** 122.

Picart-Armada, S., Thompson, W.K., Buil, A., and Perera-Lluna, A. (2018). diffuStats: an R package to compute diffusion-based scores on biological networks. *Bioinformatics* 34**,** 533-534.

Rentzsch, P., Witten, D., Cooper, G.M., Shendure, J., and Kircher, M. (2019). CADD: predicting the deleteriousness of variants throughout the human genome. *Nucleic Acids Res* 47**,** D886-D894.

Sherry, S.T., Ward, M.H., Kholodov, M., Baker, J., Phan, L., Smigielski, E.M., and Sirotkin, K. (2001). dbSNP: the NCBI database of genetic variation. *Nucleic Acids Res* 29**,** 308-311.

Shyr, C., Tarailo-Graovac, M., Gottlieb, M., Lee, J.J., Van Karnebeek, C., and Wasserman, W.W. (2014). FLAGS, frequently mutated genes in public exomes. *BMC Med Genomics* 7**,** 64.

Szklarczyk, D., Gable, A.L., Lyon, D., Junge, A., Wyder, S., Huerta-Cepas, J., Simonovic, M., Doncheva, N.T., Morris, J.H., Bork, P., Jensen, L.J., and Mering, C.V. (2019). STRING v11: protein-protein association networks with increased coverage, supporting functional discovery in genome-wide experimental datasets. *Nucleic Acids Res* 47**,** D607-D613.

Vasimuddin, M., Misra, S., Li, H., and Aluru, S. (2019). Efficient Architecture-Aware Acceleration of BWA-MEM for Multicore Systems. *IEEE International Parallel and Distributed Processing Symposium (IPDPS)***,** 314-324.
